# Ct-based diagnosis of sarcopenia as a prognostic factor for postoperative mortality after elective open-heart surgery in older patients: a cohort-based systematic review and meta-analysis

**DOI:** 10.3389/fpubh.2024.1378462

**Published:** 2024-07-08

**Authors:** Tao-Ran Yang, Peng Ji, Xiao Deng, Xi-Xia Feng, Meng-Lin He, Ru-Rong Wang, Xue-Han Li

**Affiliations:** ^1^Department of Anesthesiology, West China Hospital, Sichuan University, Chengdu, Sichuan, China; ^2^The Research Units of West China (2018RU012)-Chinese Academy of Medical Sciences, West China Hospital, Sichuan University, Chengdu, Sichuan, China; ^3^Department of Critical Care Medicine, West China Hospital, Sichuan University, Chengdu, Sichuan, China; ^4^Department of Anesthesiology, West China Second University Hospital, Sichuan University, Chengdu, Sichuan, China; ^5^Key Laboratory of Birth Defects and Related Diseases of Women and Children, Ministry of Education, Sichuan University, Chengdu, Sichuan, China; ^6^Department of Anesthesiology, Chengdu Shang Jin Nan Fu Hospital/Shang Jin Hospital of West China Hospital, Sichuan University, Chengdu, Sichuan, China

**Keywords:** sarcopenia, cardiac surgery, prognosis, skeletal muscle index, older patients

## Abstract

**Background:**

Cardiac open-heart surgery, which usually involves thoracotomy and cardiopulmonary bypass, is associated with a high incidence of postoperative mortality and adverse events. In recent years, sarcopenia, as a common condition in older patients, has been associated with an increased incidence of adverse prognosis.

**Methods:**

We conducted a search of databases including PubMed, Embase, and Cochrane, with the search date up to January 1, 2024, to identify all studies related to elective cardiac open-heart surgery in older patients. We used the Grading of Recommendations, Assessment, Development, and Evaluation (GRADE) approach to assess the certainty of evidence.

**Results:**

A total of 12 cohort studies were included in this meta-analysis for analysis. This meta-analysis revealed that patients with sarcopenia had a higher risk of postoperative mortality. Furthermore, the total length of hospital stay and ICU stay were longer after surgery. Moreover, there was a higher number of patients requiring further healthcare after discharge. Regarding postoperative complications, sarcopenia patients had an increased risk of developing renal failure and stroke.

**Conclusion:**

Sarcopenia served as a tool to identify high-risk older patients undergoing elective cardiac open-heart surgery. By identifying this risk factor early on, healthcare professionals took targeted steps to improve perioperative function and made informed clinical decisions.

**Systematic review registration**: https://www.crd.york.ac.uk/prospero/, identifier CRD42023426026.

## 1 Introduction

With the global aging population, the increasing number of comorbidities and heterogeneity in patient activity have made clinical decision-making more challenging. Over the past few decades, there was a notable rise in the level of complexity among patients undergoing cardiac open-heart surgery, corresponding with an increase in surgical risk, which warranted careful consideration and attention from healthcare providers (1, 2). The medical procedure known as open-heart surgery frequently necessitates the use of cardiopulmonary bypass, which carries a heightened risk of acute or sustained organ injury resulting from systemic inflammatory response compared to other surgical approaches (3, 4). Thus, enhancing postoperative prognosis continues to be a major concern for cardiac surgeons. However, current cardiac surgical risk scores focus primarily on the presence of specific medical comorbidities in patients and do not take into account age-related factors, particularly muscle loss (5–7). In reality, the decline in muscle or muscle mass has a significant impact on the mortality rate of older cardiovascular patients and contributes to adverse events during the perioperative period (8).

Sarcopenia is a comorbidity characterized by a progressive decline in skeletal muscle mass and strength with advancing age, commonly referred to as physical weakness. This symptom is also highly prevalent among older patients, particularly associated with adverse consequences following cardiac surgery, and results in exacerbated functional decline and elevated mortality rates (9). Despite sarcopenia’s severity, no single diagnostic criteria have been established, and most use a combination of muscle mass, muscle strength, and gait speed measurements. The most commonly used definitions are: the European Working Group on Sarcopenia in Older People [EWGSOP (2010)] (10), the revised EWGSOP2 (2019) (11), the Asian Working Group for Sarcopenia (AWGS) (12), as well as definitions using muscle mass only as a single criterion (e.g., Newman and Baumgartner definitions) (13, 14). For clinical purposes, frequently used computed tomography (CT) is an objective and quantitative diagnostic technique, which is considered as the gold standard for non-invasive assessment of muscle quantity/quality, and can quickly and easily identify sarcopenia. Some studies also believe that low muscle mass assessed by CT scan alone can diagnose sarcopenia (15–18). In patients with heart failure, skeletal muscle mass reduction can lead to reduced exercise capacity and weakness, ultimately undermining their quality of life and rehabilitation process (19). Hence, timely identification and intervention of muscle depletion are pivotal for enhancing patients’ health condition and prognosis (20).

Sarcopenia has been widely studied in relation to surgical complications across different types of surgeries. Previous studies have shown that sarcopenia is associated with various adverse outcomes in patients undergoing lung transplantation, pancreaticoduodenectomy, colorectal surgery, and liver transplantation, including prolonged mechanical ventilation, increased risk of infection, and extended hospital stay (21–25). However, there is currently no consensus on the impact of sarcopenia on mortality rate and overall condition in patients after open-heart surgery.

Although previous literature has studied the association between cardiac surgery and sarcopenia, these studies often involved different surgical approaches, including transcatheter aortic valve implantation (TAVI) or emergency surgery, which may lead to biased and inconclusive research results (26). Furthermore, there is a dearth of literature reporting the impact of sarcopenia on older patients undergoing elective cardiac open-heart surgery. Therefore, we conducted further analysis to assess the impact of sarcopenia on postoperative in-hospital mortality, and postoperative complications in older patients undergoing elective cardiac open-heart surgery.

## 2 Methods

The systematic review and meta-analysis in this study were conducted based on the Preferred Reporting Items for Systematic Reviews and Meta-Analyses (PRISMA) statement, and were registered on Prospero (registration number: CRD42023426026) (27). The whole research is quantitative analysis. Two researchers independently searched databases including PubMed, Embase, and Cochrane, with the search deadline being January 1, 2024. Furthermore, we conducted a search of the World Health Organization International Clinical Trials Registry and reviewed the bibliographies of relevant articles and reviews to identify any additional studies that potentially met the inclusion criteria, which we defined as other databases. The search was not restricted by language or region, and we have provided a PRISMA checklist. The search strategy was shown in Supplementary material 1. The review question of this manuscript was to discuss whether sarcopenia based on CT diagnosis was a prognostic factor for mortality in older patients after elective open-heart surgery.

### 2.1 Study selection

This meta-analysis aimed to include studies comparing older patients with and without sarcopenia after elective cardiac open-heart surgery. The types of studies included in the systematic review were prospective or retrospective cohort studies. Inclusion and exclusion criteria were determined prior to the start of the study. Included studies followed the PICOTS criteria: (1) population: patients with mean or median age > 60 years who underwent elective open heart surgery; (2) intervention: patients with sarcopenia were diagnosed by preoperative CT scan; (3) comparator: patients were diagnosed with non-sarcopenia before operation; (4) outcomes: the study reported the occurrence of postoperative adverse events (such as in-hospital mortality and ICU admission); (5) Timing: the time after surgery; (6) setting: Include inpatients. Our exclusion criteria were: (1) The patient underwent emergency surgery; (2) The patient had the presence of a heart implant; (3) The diagnosis of sarcopenia was unclear; (4) article types included case reports, reviews, expert opinions, or conference abstracts.

The two researchers imported the search results into citation management software (Endnote X9) and independently reviewed the titles and abstracts, selected studies that met the criteria for full-text reading, and had no knowledge of each other’s results. Any discrepancies between the researchers were resolved by a third researcher.

### 2.2 Data extraction

Two researchers independently extracted data in Endnote from eligible studies based on the Checklist for critical Appraisal and data extraction for systematic Reviews of prediction Modeling Studies (CHARMS) (28) and collected the following information: first author’s name, publication year, source of data, source of population, the characteristics of the included population, surgery type, measurement method of sarcopenia, sample size, postoperative outcome. Any discrepancies were resolved through discussion, and if necessary, another researcher was consulted.

We synthesized the data by directly extracting it from the original text. If the data was presented in the form of charts and could not be directly extracted, we used plot digitizers or contacted the corresponding author. If needed, we employed formulas provided by Hozo and other sources to convert the median and interquartile range into the mean and standard deviation (29, 30).

### 2.3 Quality assessment and risk of bias

Two researchers independently assessed the risk of bias, and disagreements were resolved through consultation with another researcher. We used the Newcastle-Ottawa Scale (NOS) to assess the quality and bias risk of cohort studies, which was a tool for critical evaluation of eligible cohort studies, mainly evaluating the quality and potential risk of bias from three aspects: selection of study population, comparability between groups, and measurement of outcomes. A score of ≥6 indicated high study quality and possible low risk of bias (31).

We utilized the GRADE approach to assess the quality of evidence for in-hospital mortality rate, complication rate, ICU length of stay, total length of hospitalization, and the number of patients requiring admission to healthcare facility after discharge. Considering factors such as risk of bias, inconsistency, imprecision, and intermittency, the evidence was categorized into high, moderate, low, and very low. We employed the GRADEpro GDT to generate the Summary of Finding (SoF) (32).

### 2.4 Outcome

The primary outcome was the in-hospital mortality of older patients after elective cardiac open-heart surgery, while the secondary outcomes included total length of hospital stay and ICU stay, the number of patients requiring admission to healthcare facility after discharge (for all causes), and outcome measures related to complications (such as the number of patients requiring continuous renal replacement therapy (CRRT), the incidence of atrial fibrillation, the incidence of pneumonia, the incidence of wound infection, the incidence of stroke, and the incidence of prolonged ventilation).

### 2.5 Data analysis

We conducted a meta-analysis using RevMan 5.3 and displayed the effect sizes of the studies using forest plots. Continuous variables were analyzed using mean differences (MD) and 95% confidence intervals, while binary variables were statistically analyzed using odds ratios. Given the heterogeneity in surgical types, surgical techniques, and operator experience, a random-effects model was employed for all the results in this study. I^2^ was used in this meta-analysis to quantifies the proportion of the variation in point estimates due to between-study differences (33). If I^2^ ≥ 50%, significant heterogeneity among studies was considered, and leave-one-out sensitivity analysis was conducted to identify potential sources of heterogeneity. Publication bias was analyzed and represented by a funnel plot, and funnel plot symmetry was assessed with Begg’s test. It was considered that there was no publication bias among the included studies when the *p*-value was >0.05 (34).

## 3 Results

**Figure 1:**
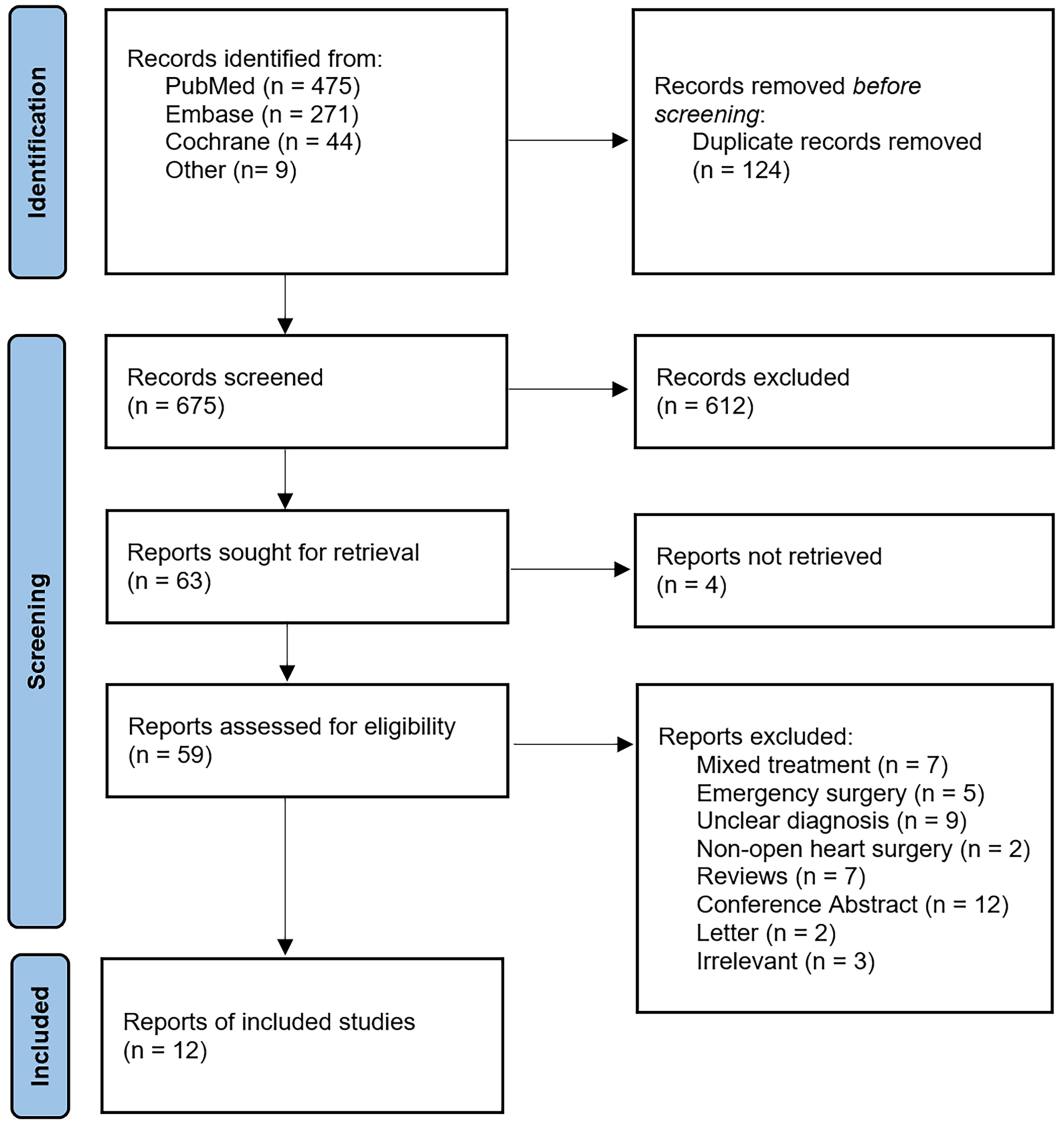
Flow chart showing selection of articles for review.

The flowchart in Figure 1 presents the process of study selection. A total of 799 studies were identified through systematic retrieval from the initial database. A total of 124 studies were excluded after removing duplicates, and after evaluating the titles and abstracts, 612 studies were excluded subsequently due to irrelevant content. Out of the remaining 63 studies, 4 articles were inaccessible in full text, leaving 59 studies for full-text reading. Following full-text screening, 47 studies were excluded due to unclear diagnosis, emergency surgery, or incompatible study types. Ultimately, a total of 12 articles were included in this study for further analysis. We conducted a quantitative analysis of the original research data reported in the 12 included studies.

Table 1 summarizes the main characteristics and details of the 12 articles that met the inclusion criteria (35–46). The total number of included patients was 4,749, with sample sizes ranging from 140 to 874, all of which were cohort studies. The majority of studies included both CABG and valve surgeries, with one study focusing on elective aortic arch replacement (38). Four studies included only valve surgeries (35, 40, 43, 44), while two studies only included patients undergoing CABG surgery (39, 46). In addition, various measurement methods for sarcopenia have been applied in different studies. A significant proportion of studies used the standardized total skeletal muscle mass normalized by the square of the height measured through CT images as the criterion for evaluating sarcopenia, known as skeletal muscle index (SMI). Moreover, certain studies employed the measurement of psoas muscle area (PMA) or psoas muscle index (PMI) to assess sarcopenia. Furthermore, one study measured grip strength and gait speed (36) while using SMI as a diagnostic criterion.

**Table 1:** Characteristics of included studies.

Author, year	Source of data	Source of population	Diagnose	Age	BMI	Surgery type	Muscle assessment method	CT measurement method	Total Sample	Sample	Postoperative outcome
Masashi 2017	Cohort study	Japan	Sarcopenia	65.5 ± 13.2	21.1 ± 3.5	CABG and Valve surgery	CT, gait speed, grip strength, 6MWD	SMI	*n* = 773	*n* = 386	Postoperative all-cause mortality					No-sarcopenia	64.4 ± 13.1	23.4 ± 3.5	*n* = 387
Ikeno 2017		Cohort study	Japan	Sarcopenia	76.2 ± 5.6				22.5 ± 3.1	Total arch replacement		CT, gait speed, grip strength	PMI	*n* = 266	*n* = 81	In-hospital mortality, Discharged to healthcare facility, Length of hospital stay, CRRT require, Atrial fibrillation, Pneumonia, Stroke, Prolonged ventilation					No-sarcopenia	75.7 ± 5.7	23.8 ± 2.9	*n* = 185
Okamura 2018	Cohort study		Japan	Sarcopenia	77.0 ± 4.6				19.6 ± 2.8					CABG and Valve surgery	CT		PMA	*n* = 428	*n* = 107	In-hospital mortality, Discharged to healthcare facility, Length of ICU stay, CRRT require, Atrial fibrillation, Stroke, Prolonged ventilation					No-sarcopenia	76.0 ± 4.3	22.6 ± 3.5	*n* = 321
Robert 2019	Cohort study	America	Sarcopenia	81 (8)	NA	Valve surgery	CT	PMI	*n* = 240		*n* = 60				Discharged to healthcare facility, Length of hospital stay, Length of ICU stay, CRRT require, Atrial fibrillation, Pneumonia, Wound infection, Stroke, Prolonged ventilation								No-sarcopenia	80 (10)	NA	*n* = 180
Yamashita 2019		Cohort study	Japan	Sarcopenia	70 ± 9.6				22.6 ± 4.0	CABG and Valve surgery or Aortic surgery	CT, gait speed, grip strength m, 6MWD	PMA	*n* = 664			*n* = 332		Postoperative all-cause mortality						No-sarcopenia	61.5 ± 14.0	22.1 ± 3.4	*n* = 332
Homare 2020			Cohort study	Japan	Sarcopenia				69.9 ± 8.9				21.6 ± 3.0	CABG		CT	PMI		*n* = 304	*n* = 76	In-hospital mortality, Discharged to healthcare facility, Length of ICU stay, CRRT require, Atrial fibrillation, Stroke, Prolonged ventilation					No-sarcopenia	66.6 ± 9.7	24.1 ± 3.3	*n* = 228
Yuriko 2020	Cohort study	Japan		Sarcopenia	75.1 ± 5.5	22.7 ± 3.4	CABG and Valve surgery	CT	PMI				*n* = 206		*n* = 63				In-hospital mortality, Discharged to healthcare facility, Length of hospital stay, Length of ICU stay, CRRT require, Atrial fibrillation, Pneumonia, Wound infection, Stroke						No-sarcopenia	73.9 ± 5.5	23.4 ± 3.7	*n* = 143
Kondo 2021		Cohort study	Japan	Sarcopenia	81.0 ± 5.8	21.6 ± 4.2				Valve surgery	CT	PMI	*n* = 140		*n* = 29			In-hospital mortality, CRRT require, Atrial fibrillation, Wound infection, Stroke, Prolonged ventilation							No-sarcopenia	77.3 ± 4.7	22.8 ± 3.7	*n* = 111
Lee 2021	Cohort study		Korea	Sarcopenia	72.17 ± 5.46	22.60 ± 2.97							Valve surgery	CT	SMI	*n* = 874	*n* = 292			30-day in-hospital events					No-sarcopenia	70.67 ± 5.47	25.29 ± 3.04	*n* = 582
Ikuko 2022		Cohort study	Japan	Sarcopenia	73.8 ± 8.8	21.5 ± 3.0	CABG and Valve surgery	CT, gait speed, grip strength	SMI							*n* = 192	*n* = 72		In-hospital mortality, Discharged to healthcare facility, Length of hospital stay, Length of ICU stay						No-sarcopenia	67.0 ± 10.1	24.5 ± 4.3	*n* = 120
Liu 2022	Cohort study		China	Sarcopenia	63.5 (18)	19.94 (2.93)				Valve surgery	CT	SMI				*n* = 216	*n* = 36	In-hospital mortality, Length of hospital stay, Atrial fibrillation, Pneumonia, Wound infection, Stroke, Prolonged ventilation							No-sarcopenia	63 (14)	24.29 (3.69)	n = 180
Shen 2023		Cohort study	China	Sarcopenia	67 (10)	22.36 (5.63)						CABG	CT	SMI	*n* = 338	n = 44	In-hospital mortality, Length of hospital stay, Length of ICU stay, CRRT require, Atrial fibrillation, Pneumonia, Wound infection, Stroke, Prolonged ventilation							No-sarcopenia	65 (11)	24.95 (3.88)	*n* = 294

The number represents the mean ± standard deviation or median (interquartile range). BMI, body mass index; NA, Not afforded; CABG, coronary artery bypass grafting; PMI, psoas muscle index; SMI, skeletal muscle index; PMA; psoas muscle area; 6MWD, 6-min walking distance; CRRT, Continuous Renal Replacement Therapy; CT, Computed Tomography.

### 3.1 Risk of bias

All the studies included in this meta-analysis were cohort studies, and the quality assessment and bias risk were conducted according to NOS. Table 2 shows the bias risk of the 12 included studies, all of which were high-quality studies (NOS score ≥ 6), suggesting a low risk of bias in the included studies.

**Table 2:** The NOS of included studies.

Study	Selection				Comparability	Outcome			Score
	Representativeness of the exposed cohort	Selection of the nonexposed cohort	Ascertainment of exposure	Demonstration that outcome of interest was not present at start of study	Comparability of Cohorts on the Basis of the Design or Analysis	Assessment of outcome	Was follow-up long enough for out comes to occur	Adequacy of follow-up of cohorts	
Masashi 2017	⭐	⭐	⭐	⭐	⭐	⭐	⭐		7
Ikeno 2017	⭐	⭐		⭐	⭐	⭐		⭐	6
Okamura 2018	⭐	⭐	⭐	⭐	⭐⭐	⭐	⭐		8
Robert 2019	⭐	⭐	⭐	⭐	⭐	⭐		⭐	7
Yamashita 2019	⭐	⭐	⭐	⭐	⭐	⭐	⭐		7
Homare 2020	⭐	⭐	⭐	⭐	⭐⭐		⭐	⭐	8
Yuriko 2020		⭐	⭐	⭐	⭐⭐	⭐	⭐		7
Kondo 2021	⭐	⭐	⭐	⭐	⭐	⭐			6
Lee 2021	⭐	⭐	⭐	⭐	⭐	⭐	⭐	⭐	8
Ikuko 2022	⭐	⭐	⭐		⭐	⭐		⭐	6
Liu 2022	⭐	⭐	⭐	⭐	⭐	⭐	⭐		7
Shen 2023	⭐	⭐	⭐	⭐	⭐⭐	⭐	⭐		8

### 3.2 Quality of evidence

**Figure 2:**
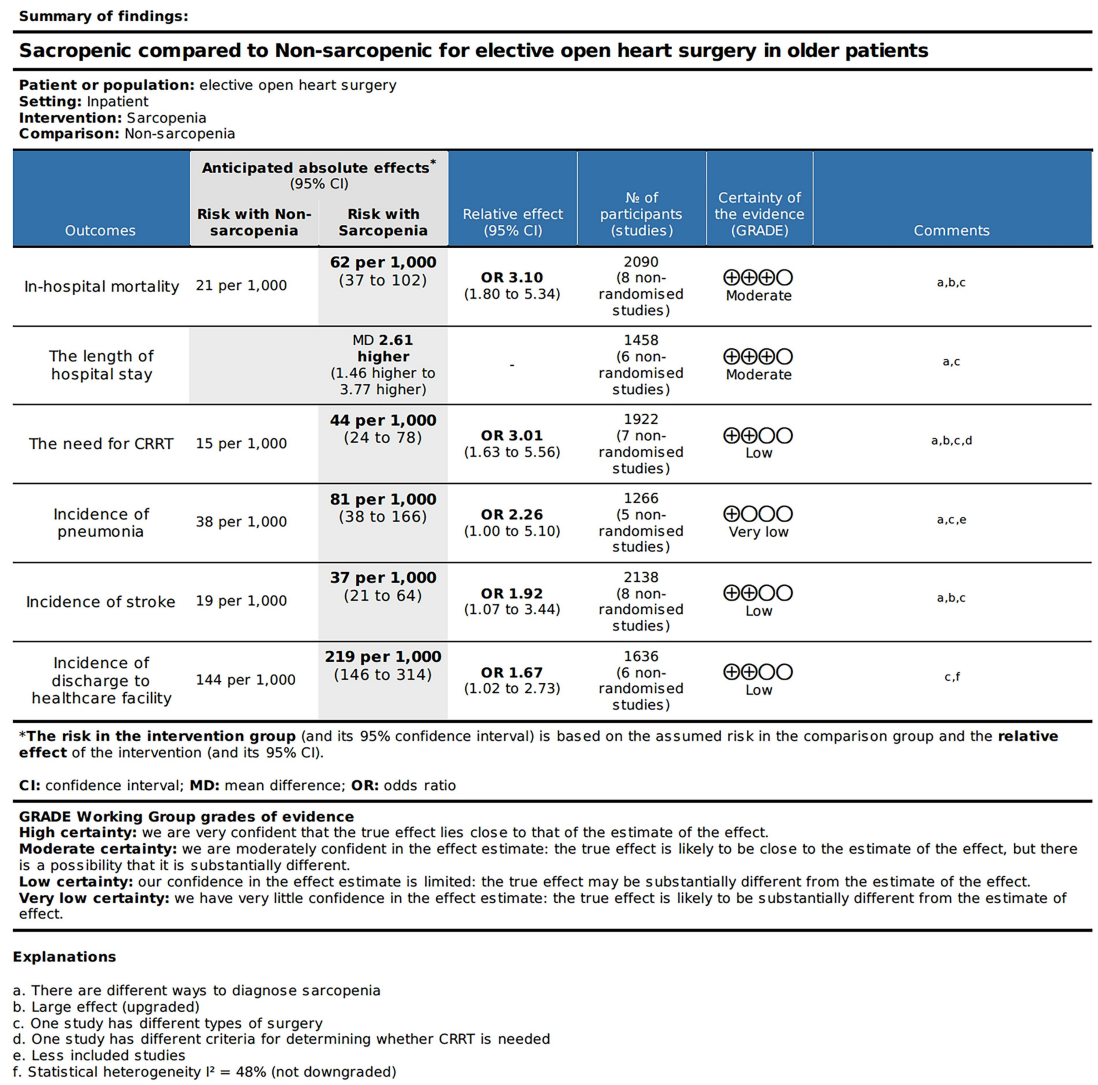
Certainty of the evidence and summary of findings.

The SOF table in Figure 2 displays the quality of evidence and relevant details for each outcome measure. Based on the GRADE approach, we found that the evidence quality for the in-hospital mortality rate, and total length of hospital stay was moderate. The evidence quality for the occurrence rate of postoperative CRRT requirement, stroke occurrence, and post-discharge healthcare facility utilization rate was relatively low. Nevertheless, the evidence quality for the occurrence rate of postoperative pneumonia was exceptionally low.

### 3.3 Primary outcome

#### 3.3.1 The relationship between sarcopenia and in-hospital mortality

**Figure 3:**
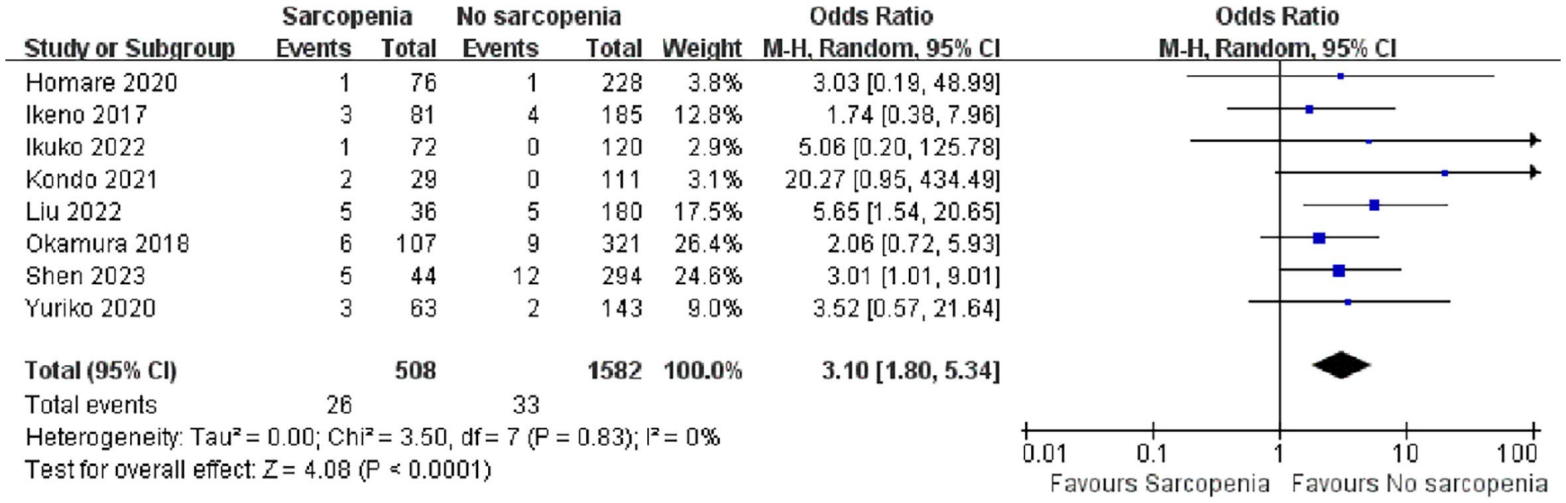
Forest plot showing the in-hospital mortality.

We conducted a quantitative analysis of eight included articles that reported in-hospital mortality, and eight articles (26–30, 34–36) reported a total of 2,090 patients with in-hospital mortality after open heart surgery. Compared with patients without sarcopenia, patients with sarcopenia had a higher risk of postoperative hospital death (Figure 3; OR: 3.10 95% CI:1.80–5.34, *p* < 0.0001). There was no heterogeneity among the included literature types (I^2^ = 0%, *p* = 0.83). In addition, we performed a publication bias analysis for primary outcomes, and funnel plots can be seen in Supplementary material 2. The Begg’s test was used for publication bias. A symmetrical appearance was checked in the funnel plot. The *p*-value of the Begg’s test for the primary outcome was 0.083 > 0.05, and no significant publication bias was found.

#### 3.3.2 Subgroup analysis of in-hospital mortality

In order to investigate the sarcopenia in depth, we conducted subgroup analysis based on different clinical characteristics included in the articles. Table 3 presents the subgroup analysis data. When conducting subgroup analysis according to the measurement methods of sarcopenia, some studies evaluated sarcopenia at vertebral level by CT scan SMI (OR: 3.98, 95% CI: 1.77–8.94) (36, 40, 46), while five studies diagnosed sarcopenia using non-SMI measurement methods (OR: 2.53, 95% CI: 1.22–5.26) (37–39, 44, 45). After analyzing the subgroup analysis based on surgical types, we confirmed that patients with sarcopenia were associated with higher in-hospital mortality regardless of including multiple surgical types or a single surgical type, with combined OR of 2.50 (95% CI: 1.04–6.01) and 3.54 (95% CI: 1.78–7.07), respectively. The population of the eight studies included are all from Asian countries, which was also consistent with the high aging society in Asian countries.

**Table 3:** The results of subgroup analysis for the In-hospital mortality.

Variable	Numbers of studies	Meta-analysis results OR (95% CI)	Heterogeneity
Measurement methods			
SMI	3	3.98 (1.77, 8.94)	I^2^ = 0%, *p* = 0.008
Non-SMI	5	2.53 (1.22, 5.26)	I^2^ = 0%, *p* = 0.01
Surgical types			
Single operation	5	3.54 (1.78, 7.07)	I^2^ = 0%, *p* = 0.0003
Multiple operations	3	2.50 (1.04, 6.01)	I^2^ = 0%, *P* = 0.04

SMI, skeletal muscle index; OR, Odds Ratio.

### 3.4 Secondary outcomes

#### 3.4.1 Number of people discharged to healthcare facility

**Figure 4:**
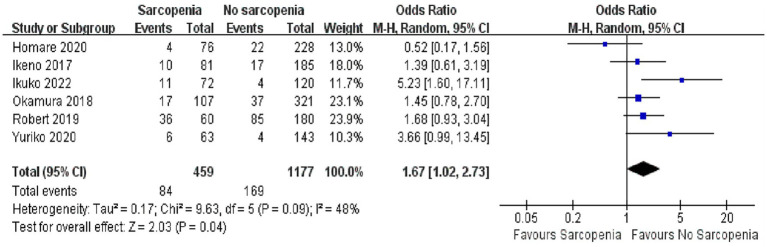
Forest plot showing the number of people discharged to healthcare facility.

In a quantitative analysis of six articles, the number of people who returned to healthcare facilities after discharge was reported, and a total of 1,636 patients were recorded (36–39, 43, 45). It was true that more patients with sarcopenia need to be admitted to healthcare facilities after surgery due to poor functional status and physical independence (Figure 4; OR: 1.67, 95% CI: 1.02–2.73, *p* = 0.04). There was some heterogeneity among the included references (I^2^ = 48%, *p* = 0.09).

#### 3.4.2 Length of hospital stay

**Figure 5:**
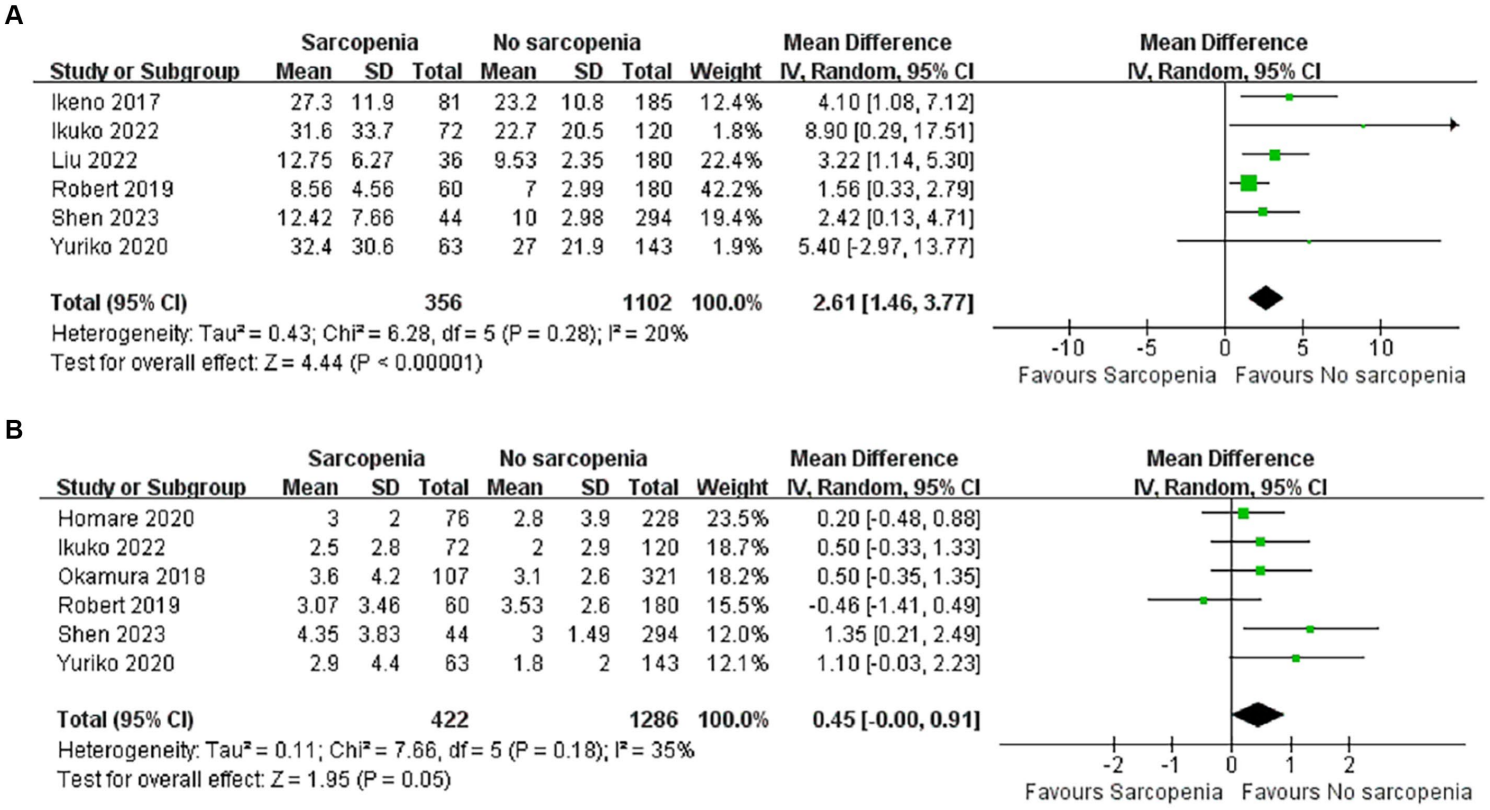
Forest plot showing the length of hospital stay and the length of ICU stay.

A total of 1,458 patients reported total length of stay after surgery in six articles (36, 38, 40, 43, 45, 46). There was a significant difference in total length of hospital stay between the sarcopenia and non-sarcopenia groups (Figure 5A; MD: 2.61, 95% CI: 1.46–3.77, *p* < 0.00001), suggesting that patients with sarcopenia remained in the hospital longer after open heart surgery. There was acceptable heterogeneity among the included references (I^2^ = 20%, *p* = 0.28).

#### 3.4.3 Length of ICU stay

There were six articles documenting length of stay in the ICU, of which 1,708 cases were reported (36, 37, 39, 43, 45, 46). Patients in the sarcopenia group had longer ICU stays (Figure 5B; MD: 0.45, 95% CI: 0.00–0.91, *p* = 0.05) and there was acceptable heterogeneity between articles (I^2^ = 35%, *p* = 0.18).

### 3.5 Secondary outcomes associated with complications

#### 3.5.1 Incidence of CRRT required

**Figure 6:**
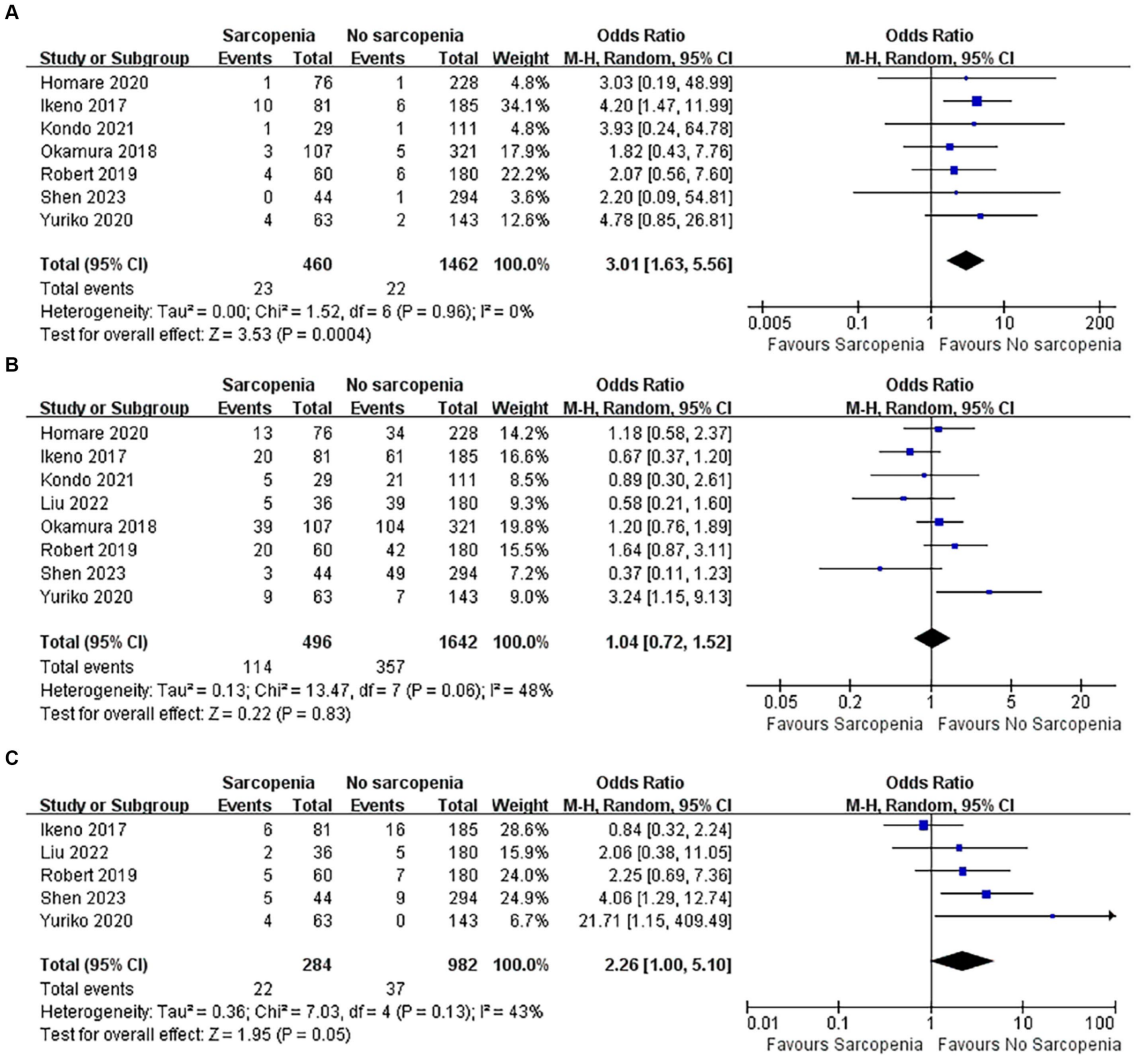
Forest plot showing the complications of part one.

There were 1,922 patients, and a total of seven articles recorded patients who required CRRT after surgery (37–39, 43–46). The number of patients with sarcopenia who required CRRT after surgery was significantly higher than that of patients without sarcopenia (Figure 6A; OR: 3.01, 95% CI: 1.63–5.56, *p* = 0.0004), indicating that there was a higher incidence of renal failure in patients with sarcopenia after cardiac open-heart surgery. There was no heterogeneity between the articles (I^2^ = 0%, *p* = 0.96).

#### 3.5.2 Incidence of postoperative atrial fibrillation

The incidence of complications after open heart surgery was quantitatively analyzed in 8 articles (37–40, 43–46), totaling 2,138 patients. There was heterogeneity among the included references (I^2^ = 48%, *p* = 0.06). Patients with sarcopenia did not have an increased incidence of new atrial fibrillation after open heart surgery (Figure 6B; OR: 1.04, 95% CI: 0.72–1.52, *p* = 0.83).

#### 3.5.3 Incidence of postoperative pneumonia

Five articles reported the incidence of pneumonia after cardiac open-heart surgery (38, 40, 43, 45, 46), including a total of 1,266 patients. There was no statistically significant difference in the incidence of postoperative pneumonia between patients with sarcopenia and those without sarcopenia (Figure 6C; OR: 2.26, 95% CI: 1.00–5.10, *p* = 0.05). There was acceptable heterogeneity among the included studies (I^2^ = 43, *p* = 0.13).

#### 3.5.4 Incidence of postoperative wound infection

**Figure 7:**
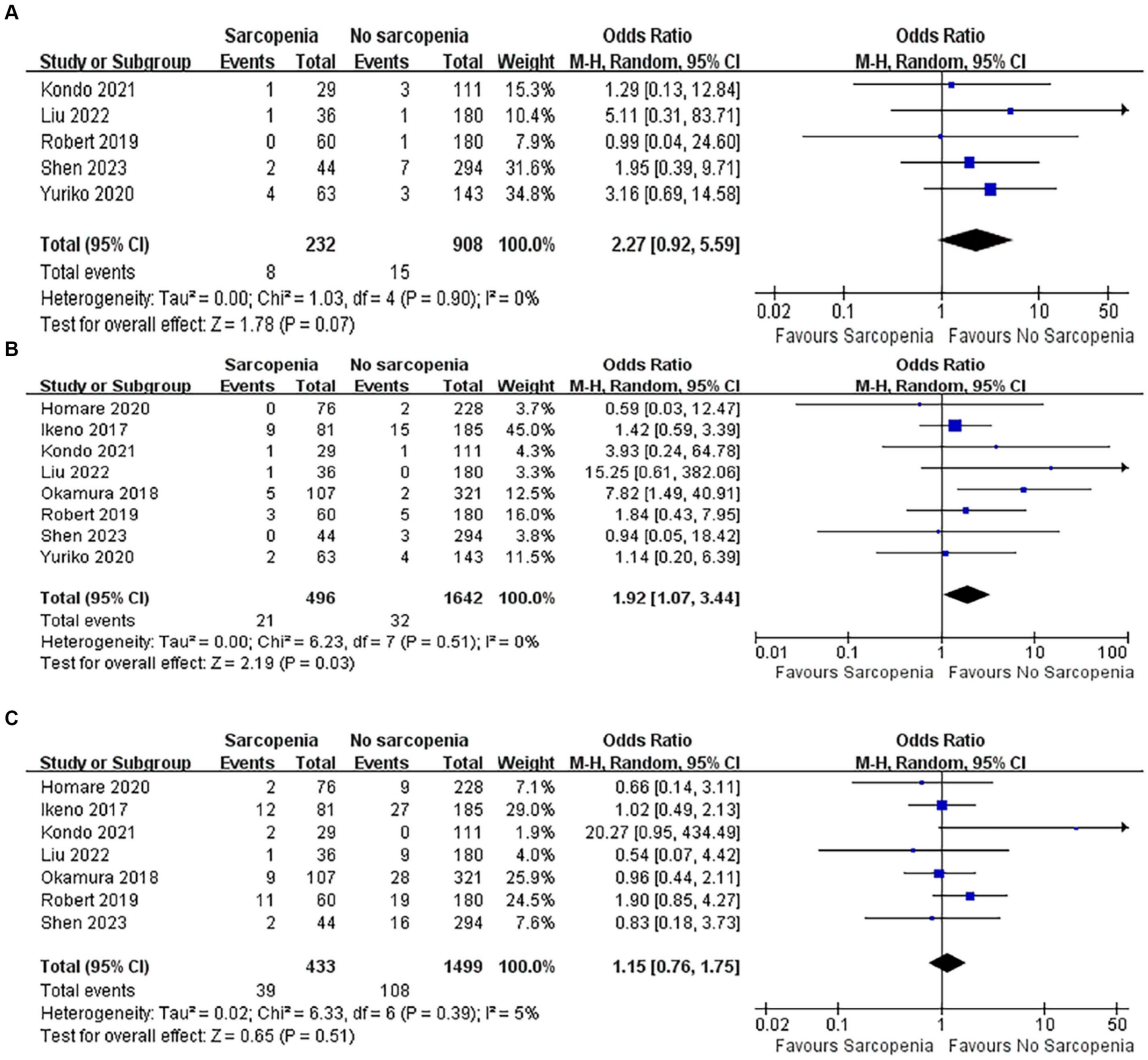
Forest plot showing the complications of part two.

Five articles with a total of 1,140 patients of postoperative wound infections were quantitatively analyzed (40, 43–46). There was no difference in the incidence of wound infection between the sarcopenia group and the non-sarcopenia group (Figure 7A; OR: 2.27, 95% CI: 0.92–5.59, *p* = 0.07), and there was no heterogeneity between the articles (I^2^ = 0%, *p* = 0.90).

#### 3.5.5 Incidence of postoperative stroke

Eight articles quantitatively reported the incidence of stroke in 2,138 patients after open heart surgery (37–40, 43–46). Compared with patients without sarcopenia, patients with sarcopenia had a higher risk of postoperative stroke (Figure 7B; OR: 1.92, 95% CI: 1.07–3.44, *p* = 0.03). There was no heterogeneity among the included literature types (I^2^ = 0%, *p* = 0.51).

#### 3.5.6 Incidence of postoperative prolonged ventilation

There were seven articles quantitatively reporting the relationship between sarcopenia and the incidence of prolonged postoperative ventilation (37–40, 43, 44, 46). The results showed that patients with sarcopenia did not have an increased incidence of prolonged postoperative ventilation (Figure 7C; OR: 1.15, 95% CI: 0.76–1.75). The included original literature had low heterogeneity (I^2^ = 5%, *p* = 0.39).

## 4 Discussion

With increasing life expectancy and an aging population, the prevalence of sarcopenia has increased, and the proportion of patients with sarcopenia undergoing heart surgery is increasing (10, 47). However, there is a scarcity of studies investigating the effects of sarcopenia on open heart surgery. To address this gap, this study sought to investigate postoperative mortality in patients with preoperative sarcopenia. Through a rigorous systematic review and meta-analysis, we analyzed a total of 12 articles encompassing 4,749 patients. The results indicated that older patients with sarcopenia had higher postoperative mortality. Furthermore, patients with sarcopenia had longer stays in ICU, longer total hospital stays, increased need for postoperative CRRT, and heightened incidence of postoperative complications.

Patients diagnosed with sarcopenia prior to surgery were also associated with higher postoperative mortality, and there was no heterogeneity between studies. We performed a series of subgroup analyses based on the method of sarcopenia measurement, and type of surgery. Subgroup analysis showed that sarcopenia was significantly associated with higher postoperative mortality across different surgical modalities, and even subgroups of different sarcopenia measurement methods, which may have been due to the high negative impact of sarcopenia, which is associated with adverse outcomes in any subgroup. In certain subgroups, high heterogeneity could cause bias between aggregated results and actual results, which may need further confirmation.

Although previous studies have reported that preoperative sarcopenia defined from the psoas region was associated with a greater risk of long-term mortality and major unscrupulous cerebrovascular events in older patients undergoing heart surgery (48). However, there are still some cardiac studies, in order to predict the incidence of postoperative pulmonary complications, the chest muscle is used to calculate the SMI index. This may be a possible source of heterogeneity in this study.

Our systematic review and meta-analysis independently investigated the association between sarcopenia and outcomes in older patients following elective cardiac open-heart surgery. Recently, the impact of sarcopenia on cardiac surgery has received extensive attention, and in cardiovascular surgery, sarcopenia is the most influential factor in slowing the progress of cardiac rehabilitation and increasing postoperative complications (49, 50). Previous studies on the effects of sarcopenia on TAVI have concluded that sarcopenia was closely associated with mortality and adverse outcomes after multiple surgeries, but no systematic article evaluated the relationship between sarcopenia and elective cardiac open-heart surgery. Cardiac open-heart surgery is always accompanied by cardiopulmonary bypass, which requires the opening of the chest for surgical procedures, and such patients are at a higher risk of acute or persistent sexual organ damage due to trauma and systemic inflammation than patients undergoing other surgeries. We therefore conducted a review of the topic and used GRADE to assess the quality of evidence in included studies. In our study, the research heterogeneity was low, the sample size was large, and the methodology was reliable, which increased the reliability and representativeness of the conclusions.

This meta-analysis included only older patients who had elective open-heart surgery. Therefore, in the conclusion of this study, the identification of sarcopenia before surgery may provide better medical management strategies and targets when it is found that sarcopenia may lead to adverse postoperative outcomes in patients undergoing this type of surgery. Introducing sarcopenia into a risk assessment can provide a better understanding of a patient’s ability to tolerate surgery and help guide patients to more appropriate forms of treatment. For patients with sarcopenia, preoperative exercise training and nutritional supplements can be taken. Active rehabilitation programs can promote early postoperative activity, reduce the associated frequency of morbidity, and further improve activity throughout the life course.

Although these meta-analyses bring together evidence and illustrate that sarcopenia is a key factor of prognostic value in cardiac open-heart surgery, there are some limitations to this study. First, the 12 studies included were cohort studies, some of which measured sarcopenia differently. Some studies used the psoas muscle area or psoas muscle index to diagnose sarcopenia. Although psoas muscle has been recognized and recommended by some studies to diagnose sarcopenia (51–54), some literature has questioned this (55–57), and EWSOP2 still considers psoas as a small muscle, which may not reflect the state of the whole muscle (11). It is recommended to use the skeletal muscle index of the total lumbar muscle area (at the level of the third lumbar vertebra) to diagnose sarcopenia (58). Although the results of this study indicated that regardless of the measurement method used, the in-hospital mortality rate in the sarcopenia group was higher than the non-sarcopenia group, the inconsistency in measurement methods may pose a risk of inaccurately diagnosing sarcopenia using psoas muscle, potentially leading to biased results. Secondly, the definition of sarcopenia lacks a unified standard, which may be due to differences in human populations. Some original studies only use CT scans to diagnose sarcopenia without evaluating the patients’ muscle strength, neglecting the potential impact of muscle strength, which could introduce bias in the diagnosis of sarcopenia and even lead to deviations in postoperative physical interventions for these patients, resulting in unfavorable outcomes. Future research should not only assess muscle mass/quantity reduction through CT scans but also include evaluations of patients’ muscle strength to better and more accurately identify various aspects of sarcopenia. Furthermore, some patients in the included literature may have a history of cardiac open-heart surgery in the past, and multiple thoracotomies will increase the incidence of adverse events in patients. In addition, positive results are more likely to be published, and there may be a risk of reporting bias. Therefore, more clinical studies in multiple centers are needed to confirm the value of sarcopenia in open heart surgery, and to conduct early targeted intervention for sarcopenia to improve the prognosis of patients.

## 5 Conclusion

This systematic review and meta-analysis found that sarcopenia diagnosed by preoperative CT scan was associated with higher rates of in-hospital mortality and complications in older patients after elective cardiac open-heart surgery, as well as significantly higher ICU and total length of stay.

We recommend that sarcopenia should be included in the routine evaluation of patients undergoing elective cardiac open-heart surgery, which may help clinicians refine treatment strategies and improve short - and long-term outcomes for patients.

## Data availability statement

The original contributions presented in the study are included in the article/Supplementary material, further inquiries can be directed to the corresponding author.

## Author contributions

T-rY: Writing – original draft, Software, Methodology, Conceptualization. PJ: Writing – original draft, Validation, Software, Data curation. XD: Writing – original draft, Data curation. X-xF: Writing – original draft, Data curation. M-lH: Writing – original draft, Software. R-rW: Writing – review & editing, Supervision. X-hL: Writing – review & editing, Supervision, Methodology, Formal analysis, Conceptualization.
