# Supplementary material for: Ct-based diagnosis of sarcopenia as a prognostic factor for postoperative mortality after elective open-heart surgery in older patients: a cohort-based systematic review and meta-analysis
Source: Front Public Health. 2024 Jul 8;12:1378462. doi: 10.3389/fpubh.2024.1378462 (PMC11261807; doi:10.3389/fpubh.2024.1378462)
Supplement: Supplementary file 1 [file Data_Sheet_1.docx]

Supplementary Material

# Supplementary Table

PubMed

| Search | Query |  |  |  |  |  |  |  |  |  |  |  |
| --- | --- | --- | --- | --- | --- | --- | --- | --- | --- | --- | --- | --- |
| #1 | " Cardiovascular Surgical Procedures "[MeSH Terms] OR " Surgical Procedure, Cardiovascular "[All Fields] OR " Procedure, Cardiovascular Surgical "[All Fields] | | | | | | | | | | | |
| #2 | "Cardiac Surgical Procedures"[MeSH Terms] OR "Procedure, Cardiac Surgical"[All Fields] OR "Heart Surgical Procedures"[All Fields] | | | | | | | | | | | |
| #3 | #1 OR #2 | | | | | | | | | | | |
| #4 | "Open"[All Fields] | | | | | | | | | | | |
| #5 | #3 AND #4 | | | | | | | | | | | |
| #6 | “Aged" [MeSH Terms] OR "Aged" [All Fields] OR "Older people" [All Fields] OR "Elderly [All Fields]" | | | | | | | | | | | |
| #7 | #5 AND #6 | | | | | | | | | | | |
| #8 | "Elective Surgical Procedures"[MeSH Terms] OR "Surgical Procedures, Elective"[All Fields] | | | | | | | | | | | |
| #9 | #7 AND #8 | | | | | | | | | | | |
| #10 | "Sarcopenia"[MeSH Terms] OR "Sarcopenias"[All Fields] | | | | | | | | | | | |
| #11 | #9 AND #10 | | | | | | | | | | | |

Embase

| Search | Query |  |  |  |  |  |  |  |  |  |  |  |
| --- | --- | --- | --- | --- | --- | --- | --- | --- | --- | --- | --- | --- |
| #1 | 'Sarcopenia'/exp OR 'Sarcopenia' | | | | | | | | | | | |
| #2 | 'Heart'/exp OR 'Heart' | | | | | | | | | | | |
| #3 | 'Surgical procedures, operative'/exp OR 'Surgical procedures, operative' OR (surgical AND ('procedures,'/exp OR procedures,) AND operative) | | | | | | | | | | | |
| #4 | 'Cardiac surgical procedures'/exp OR 'Cardiac surgical procedures' OR (('cardiac'/exp OR cardiac) AND surgical AND ('procedures'/exp OR procedures)) | | | | | | | | | | | |
| #5 | 'Open'/exp OR 'Open' | | | | | | | | | | | |
| #6 | 'Aged'/exp OR 'Aged' OR 'Elderly'/exp OR 'Elderly' | | | | | | | | | | | |
| #7 | 'Elective Surgical Procedures'/exp OR 'Elective Surgical Procedures' | | | | | | | | | | | |
| #8 | #2 AND #3 | | | | | | | | | | | |
| #9 | #4 OR #8 | | | | | | | | | | | |
| #10 | #5 AND #9 | | | | | | | | | | | |
| #11 | #6 AND #10 | | | | | | | | | | | |
| #12 | #7 AND #11 | | | | | | | | | | | |
| #13 | #1 AND #12 | | | | | | | | | | | |

Cochrane library

| Search | Query |  |  |  |  |  |  |  |  |  |  |  |
| --- | --- | --- | --- | --- | --- | --- | --- | --- | --- | --- | --- | --- |
| #1 | Mesh descriptor: [Sarcopenia] explode all trees | | | | | | | | | | | |
| #2 | Mesh descriptor: [Heart] explode all trees | | | | | | | | | | | |
| #3 | Mesh descriptor: [Surgical procedures, operative] explode all trees | | | | | | | | | | | |
| #4 | Mesh descriptor: [Cardiac surgical procedures] explode all trees | | | | | | | | | | | |
| #5 | Mesh descriptor: [Open] explode all trees | | | | | | | | | | | |
| #6 | Mesh descriptor: [Aged] explode all trees | | | | | | | | | | | |
| #7 | Mesh descriptor: [Elective Surgical Procedures] explode all trees | | | | | | | | | | | |
| #8 | Sarcopenia | | | | | | | | | | | |
| #9 | Heart | | | | | | | | | | | |
| #10 | "Operative Procedure" OR "Procedure, Operative" OR "Surgical Procedure, Operative" OR "Operative Surgical Procedures" OR "Procedure, Operative Surgical" OR "Surgical Procedures" OR "Procedure, Surgical" | | | | | | | | | | | |
| #11 | "Procedure, Cardiac Surgical" OR "Surgical Procedure, Cardiac" OR "Surgical Procedures, Heart" OR "Cardiac Surgical Procedure" OR "Heart Surgical Procedures" OR "Procedure, Heart Surgical" OR "Surgical Procedures, Heart" OR "Surgery, Cardiac" | | | | | | | | | | | |
| #12 | Open | | | | | | | | | | | |
| #13 | "Aged" OR "Older people" OR "Elderly" | | | | | | | | | | | |
| #14 | "Elective Surgical Procedures" OR "Surgical Procedures, Elective | | | | | | | | | | | |
| #15 | #1 OR #8 | | | | | | | | | | | |
| #16 | #2 OR #9 | | | | | | | | | | | |
| #17 | #3 OR #10 | | | | | | | | | | | |
| #18 | #4 OR #11 | | | | | | | | | | | |
| #19 | #5 OR #12 | | | | | | | | | | | |
| #20 | #6 OR #13 | | | | | | | | | | | |
| #21 | #7 OR #14 | | | | | | | | | | | |
| #22 | #16 AND #17 | | | | | | | | | | | |
| #23 | #18 OR #22 | | | | | | | | | | | |
| #24 | #19 AND #23 | | | | | | | | | | | |
| #25 | #20 AND #24 | | | | | | | | | | | |
| #26 | #21 AND #25 | | | | | | | | | | | |
| #27 | #1 AND #26 | | | | | | | | | | | |

# Supplementary Figure





**Supplementary 2** Funnel plot of publication bias for meta-analysis of the relationship between sarcopenia and in-hospital mortality.
